# Readmission Rates and Episode Costs for Alzheimer Disease and Related Dementias Across Hospitals in a Statewide Collaborative

**DOI:** 10.1001/jamanetworkopen.2023.2109

**Published:** 2023-03-16

**Authors:** Neil Kamdar, John Syrjamaki, James E. Aikens, Elham Mahmoudi

**Affiliations:** 1 Institute for Healthcare Policy and Innovation, University of Michigan Medical School, Ann Arbor; 2 Department of Surgery, University of Michigan Medical School, Ann Arbor; 3 Department of Family Medicine, University of Michigan Medical School, Ann Arbor; 4 Department of Physical Medicine and Rehabilitation, University of Michigan Medical School, Ann Arbor; 5 Center for Population Health Sciences, Stanford University, Stanford, California; 6 Michigan Value Collaborative, University of Michigan Medical School, Ann Arbor

## Abstract

**Importance:**

There has been a paucity of research examining the risk and cost of readmission among patients with Alzheimer disease and related dementias (ADRD) after a planned hospitalization for a broad set of medical and surgical procedures.

**Objective:**

To examine 30-day readmission rates and episode costs, including readmission costs, for patients with ADRD compared with their counterparts without ADRD across Michigan hospitals.

**Design, Setting, and Participants:**

This retrospective cohort study used 2012 to 2017 Michigan Value Collaborative data across different medical and surgical services stratified by ADRD diagnosis. A total of 66 676 admission episodes of care that occurred between January 1, 2012, and June 31, 2017, were identified for patients with ADRD using *International Classification of Diseases, Ninth Revision, Clinical Modification *(*ICD-9-CM*) and *International Statistical Classification of Diseases, Tenth Revision, Clinical Modification *(*ICD-10-CM*) diagnostic codes for ADRD, along with 656 235 admission episodes in patients without ADRD. Using a generalized linear model framework, this study risk adjusted, price standardized, and performed episode payment winsorization. Payments were risk adjusted for age, sex, Hierarchical Condition Categories, insurance type, and prior 6-month payments. Selection bias was accounted for using multivariable logistic regression with propensity score matching without replacement using calipers. Data analysis was performed from January to December 2019.

**Exposure:**

Presence of ADRD.

**Main Outcomes and Measures:**

Main outcomes were 30-day readmission rate at the patient and county levels, 30-day readmission cost, and 30-day total episode cost across 28 medical and surgical services.

**Results:**

The study included 722 911 hospitalization episodes, of which 66 676 were related to patients with ADRD (mean [SD] age, 83.4 [8.6] years; 42 439 [63.6%] female) and 656 235 were related to patients without ADRD (mean [SD] age, 66.0 [15.4] years; 351 246 [53.5%] female). After propensity score matching, 58 629 hospitalization episodes were included for each group. Readmission rates were 21.5% (95% CI, 21.2%-21.8%) for patients with ADRD and 14.7% (95% CI, 14.4%-15.0%) for patients without ADRD (difference, 6.75 percentage points; 95% CI, 6.31-7.19 percentage points). Cost of 30-day readmission was $467 higher (95% CI of difference, $289-$645) among patients with ADRD ($8378; 95% CI, $8263-$8494) than those without ($7912; 95% CI, $7776-$8047). Across all 28 service lines examined, total 30-day episode costs were $2794 higher for patients with ADRD vs patients without ADRD ($22 371 vs $19 578; 95% CI of difference, $2668-$2919).

**Conclusions and Relevance:**

In this cohort study, patients with ADRD had higher readmission rates and overall readmission and episode costs than their counterparts without ADRD. Hospitals may need to be better equipped to care for patients with ADRD, especially in the postdischarge period. Considering that any type of hospitalization may put patients with ADRD at a high risk of 30-day readmission, judicious preoperative assessment, postoperative discharge, and care planning are strongly advised for this vulnerable patient population.

## Key Points

**Question:**

What are the differences in readmission rates and episode costs between patients with Alzheimer disease and related dementias (ADRD) and patients without ADRD?

**Findings:**

This cohort study of 722 911 hospitalization episodes found that patients with ADRD had significantly higher readmission rates than the general geriatric population (21.5% vs 14.7%). The total 30-day episode cost of hospitalization was also higher among patients with ADRD than for patients without ($22 371 vs $19 578), associated with postdischarge costs.

**Meaning:**

The higher readmission rates and higher costs suggest that patients with ADRD should undergo careful preoperative assessment to assess their fitness for medical or surgical treatment, and hospitals should be better prepared to address the needs of hospitalized patients with ADRD.

## Teaser

This cohort study compares 30-day readmission rates, 30-day readmission costs, and total episode costs among patients with vs without Alzheimer disease and related dementias.

## Introduction

The prevalence of Alzheimer disease and related dementias (ADRD) is estimated to double from 1.6% of the population in 2014 to 3.3% (13.9 million Americans) by 2060.^1^ Older people are commonly admitted to acute care hospitals, and an increasing number of them will have ADRD.^2^ In 2013, the rate of unplanned 30-day readmission among patients with ADRD was 18.2% compared with 17.3% among the general geriatric population.^2^ The higher rates of readmission and other adverse events might indicate that hospitals are insufficiently equipped to handle patients with ADRD.^3,4,5^ Hospital discharges often come without adequate preparation for complex case management needs of patients with ADRD and their caregivers.^6^ Furthermore, although readmissions may have detrimental consequences for all older patients, adverse events, such as readmissions, would put patients with ADRD at a much higher risk for delirium, further cognitive decline, institutionalization, and premature death.^2^

Research indicates that the costs of hospitalization are 3 times higher ($11 540 vs $3391) and postacute care is 9 times higher ($6755 vs $727) for patients with ADRD vs their counterparts.^7^ Although some hospitalizations are unavoidable and necessary, unplanned readmissions are a non–value-added burden to patients with ADRD, their caregivers, health care practitioners, and health systems.^8,9^ With an estimated annual cost of $4.7 billion,^2^ readmissions and potentially preventable hospitalizations substantially increase the cost of care for patients with ADRD.^8,10,11^ There is a paucity of research on risk and episode costs of readmission among patients with ADRD compared with the general geriatric population.^12,13^ In addition, it is not known which of the surgical inpatient services has a higher risk of readmission and whether the risk of readmission among these services is different between patients with and without ADRD.

In this study, we addressed these gaps by using 2012 to 2017 data from the Michigan Value Collaborative (MVC).^14^ The 3 main aims of this study were to examine (1) 30-day readmission rate at the episode and county levels, (2) 30-day readmission cost, and (3) total episode costs. All 3 aims were examined across 28 different service lines or surgical procedures and stratified by patients’ diagnosis of ADRD.

## Methods

### Data Source

The MVC includes 100 acute care hospitals in Michigan (77 included at the time of the study).^14^ The MVC is a partnership between Michigan hospitals and the state’s largest commercial health care insurer (Blue Cross Blue Shield of Michigan [BCBSM]). The MVC is a collaborative quality initiative program whose one of many aims is to understand variation in health care use and costs and define best clinical practices during the perioperative hospitalization period. The MVC develops and maintains a claims-based registry of payments and utilization for 30- and 90-day episodes of care for BCBSM’s preferred provider organizations (PPOs) and Medicare fee-for-service (FFS) patients. The MVC risk adjusts total episode payments as well as component payments using generalized linear regression models, adjusting for patient characteristics, 79 Hierarchical Condition Categories, and prior 6-month payments before index hospitalization reflected by the service line to calculate expected payments.^15^ Furthermore, all 30-day and episode payments were price standardized according to the Medicare fee schedule, with consistent methods as previously described by the *Dartmouth Atlas of Healthcare*,^16^ in which mean Medicare payments within the state account for differences attributable to payer type, inflation, regional variation, and contractual differences between payers and hospitals or health care systems. Price standardization is performed for all payments and adjusted for inflation based on the Consumer Price Index to reflect the most recent year’s dollars in the study (2017). Details of the price standardizations and risk adjustment algorithms are available in the eAppendix 1 and eAppendix 2 in Supplement 1. This cohort study was deemed exempt by the University of Michigan Medical School Institutional Review Board because all patient data were deidentified at the time they were obtained and posed no patient privacy risks. We followed the Strengthening the Reporting of Observational Studies in Epidemiology (STROBE) reporting guideline (eFigure 1 in Supplement 1).

### Patient Selection

Our cases include patients 65 years and older with evidence of ADRD diagnosis using any claim throughout their enrollment history through the *International Classification of Diseases, Ninth Revision, Clinical Modification *(*ICD-9-CM*) and *International Statistical Classification of Diseases, Tenth Revision, Clinical Modification *(*ICD-10-CM*) codes who had at least 1 hospitalization episode of any type throughout their enrollment with a BCBSM plan or Medicare FFS (eTable 1 in Supplement 1). As is common with many claims data sets, race and ethnicity as well as other identifying characteristics, such as sociodemographic information, are not available. Hospitalization episodes for each of the service lines that represent medical and surgical hospitalizations were categorized using *Current Procedural Terminology* or *ICD-9-CM* or *ICD-10-CM* codes.^14^ We selected all episodes of care between January 1, 2012, and June 31, 2017, that received reimbursement from BCBSM PPOs or Medicare FFS. A schematic flow diagram of the sample size of the final cohort selection is presented in eFigure 1 in Supplement 1.

### Outcome Variables

Our primary outcomes were 30-day readmission rate, 30-day readmission cost, and 30-day total episode costs. Total episode costs (payments) were divided into the following components: total professional episode, index hospitalization, 30-day readmission, total post discharge, total outpatient home health, inpatient rehabilitation, and skilled nursing facility. Hospitalization readmission payments were reported for patients with and without ADRD who had a 30-day readmission.

### Matching Algorithm

To account for selection bias attributable to differences in age, sex, comorbidities (using 79 Hierarchical Condition Categories), 28 different types of medical or surgical procedures, and insurance type, we performed propensity score matching via multivariable logistic regression with a caliper size of 0.0001 using a 1:1 ratio without replacement matching algorithm (Table 1). Because analyses involved large sample sizes, we assessed clinical significance using standardized differences based on calculating the Cohen *d* or *h* statistic.^17^ Clinically and statistically meaningful standardized differences were considered at a cutoff of 0.2 or higher.

**Table 1.** Prematched and Postmatched Cohort Baseline Characteristic Differences Between Patients With and Without ADRD^a^

Characteristics	Prematched cohort (service lines)			Postmatched cohort (service lines)		
	Without ADRD (n = 656 235)	With ADRD (n = 66 676)	Standardized difference^b^	Without ADRD (n = 58 629)	With ADRD (n = 58 629)	Standardized difference^b^
Age, mean (SD), y	66.0 (15.4)	83.4 (8.6)	1.17	82.9 (8.9)	82.7 (8.6)	0
Sex						
Female	351 246 (53.5)	42 439 (63.6)	0.21	36 977 (63.9)	36 779 (62.7)	0
Male	304 989 (46.5)	24 237 (36.4)		21 652 (36.1)	21 850 (37.3)	0
Michigan Value Collaborative service lines						
Atrial fibrillation	38 448 (5.9)	4780 (7.2)	0.05	4555 (7.8)	4508 (7.7)	0
Acute myocardial infarction	48201 (7.3)	6599 (9.9)	0.09	6167 (10.5)	6115 (10.4)	0
Appendectomy	11 353 (1.7)	136 (0.2)	0.17	119 (0.2)	135 (0.2)	0.01
Coronary artery bypass graft	8948 (1.4)	250 (0.4)	0.11	253 (0.4)	250 (0.4)	0
Congestive heart failure	68 139 (10.4)	13 057 (19.6)	0.26	12 753 (21.8)	12 149 (20.7)	0.03
Cholecystectomy	39 173 (6.0)	877 (1.3)	0.26	751 (1.3)	857 (1.5)	0.02
Colectomy						
Noncancerous	14580 (2.2)	951 (1.4)	0.06	899 (1.5)	903 (1.5)	0
Cancerous	5091 (0.8)	286 (0.4)	0.05	300 (0.5)	278 (0.5)	0.01
Disk herniation	7806 (1.2)	43 (0.1)	0.17	35 (0.1)	43 (0.1)	0.01
Endarterectomy	8725 (1.3)	333 (0.5)	0.09	360 (0.6)	332 (0.6)	0.01
Esophagectomy	136 (<0.1)	5 (<0.1)	0.01	8 (<0.1)	5 (<0.1)	0
Gastrectomy	139 (<0.1)	4 (<0.1)	0.01	6 (<0.1)	4 (<0.1)	0
Hernia repair						
Abdominal	25 038 (3.8)	295 (0.4)	0.26	239 (0.4)	294 (0.5)	0.01
Groin	24 265 (3.7)	495 (0.7)	0.21	458 (0.8)	490 (0.8)	0.01
Hip fracture	13 085 (2.0)	7640 (11.5)	0.41	5635 (9.6)	5810 (9.9)	0.01
Hysterectomy	27 418 (4.2)	117 (0.2)	0.33	85 (0.1)	117 (0.2)	0.01
Joint hip and knee replacement	95 606 (14.6)	1449 (2.2)	0.49	1307 (2.2)	1447 (2.5)	0.02
Kidney stone injury	32 081 (4.9)	731 (1.1)	0.24	601 (1.0)	712 (1.2)	0.02
Lung cancer resection	2824 (0.4)	76 (0.1)	0.06	71 (0.1)	76 (0.1)	0
Other spine surgery	33 840 (5.2)	737 (1.1)	0.25	701 (1.2)	734 (1.3)	0.01
Other trauma procedure	53 496 (8.2)	16 555 (24.8)	0.46	13 168 (22.5)	13 516 (23.1)	0.01
Pancreatectomy	409 (0.1)	11 (<0.1)	0.02	16 (<0.1)	11 (<0.1)	0.01
PCI	32 889 (5.0)	748 (1.1)	0.24	690 (1.2)	746 (1.3)	0.01
Prostatectomy	5369 (0.8)	24 (<0.1)	0.14	22 (<0.1)	24 (<0.1)	0
Roux-en-y gastric bypass	3074 (0.5)	6 (<0.1)	0.12	4 (<0.1)	6 (<0.1)	0
Gastric sleeve	9668 (1.5)	9 (<0.1)	0.22	6 (<0.1)	9 (<0.1)	0
Stroke	36 583 (5.6)	9943 (14.9)	0.32	8873 (15.1)	8542 (14.6)	0.02
Valve	9851 (1.5)	519 (0.8)	0.07	547 (0.9)	516 (0.9)	0.01

Abbreviations: ADRD, Alzheimer disease and related dementias; PCI, percutaneous coronary intervention.

^a^ Data are presented as number (percentage) of patients unless otherwise indicated. Data are from the 2012-2017 Michigan Value Collaborative.

^b^ Standardized difference calculations examined meaningful differences in baseline characteristics before and after propensity matching. Cohen *d* and *h* statistics were calculated for numerical and categorical variables, respectively. Standardized differences of 0.2 or greater were considered meaningful differences between both groups.

### Statistical Analysis

Geographic unadjusted readmission rates for counties with at least 1 hospital represented in the state were compared between patients with and without ADRD. Readmission rates for patients with and without ADRD were split into quartiles. Statewide county-level heat maps (choropleth) of ADRD and non-ADRD episodes were analyzed to qualitatively examine countywide variation in readmission.

Using observed-to-expected methods previously described by the Centers for Medicare & Medicaid Services (CMS) and the Agency for Healthcare Research and Quality,^18^ we computed the observed-to-expected ratios and standardized them via multiplying by the reference mean observed payments across all episodes for each specific payment component. This method enabled us to obtain the risk-adjusted and price-standardized episode payment. To account for right skewness in the distribution of episode payments, we performed winsorization at the 99th and 1st percentiles, consistent with other episode-payment analysis conducted within the MVC.^19^ Winsorization is an approach that assigns the extreme outliers to the 99th and 1st percentiles such that statistical group comparisons can assume normality in their distribution and parametric methods are sufficiently robust. Postmatched episode payments were compared between patients with and without ADRD across and within service lines, with more than 20 episodes in each group.

Because differences in cost between patients with and without ADRD can be attributable to initial treatment cost differences, differences in probabilities of readmission, and differences in cost of readmission, we used a decomposition framework for each procedure. We calculate the procedure-specific difference in mean total 30-day risk-adjusted and price-standardized episode payments for patients with and without ADRD. Details of this approach are outlined in eAppendix 3 in Supplement 1.

All analyses were conducted using SAS software, version 9.4 (SAS Institute Inc) with standardized difference cutoffs at 0.2 or higher to classify meaningful differences and reported 95% CIs. Data analysis was performed from January to December 2019.

## Results

The study included 722 911 hospitalization episodes, of which 66 676 were related to patients with ADRD (mean [SD] age, 83.4 [8.6] years; 42 439 [63.6%] female) and 656 235 were related to patients without ADRD (mean [SD] age, 66.0 [15.4] years; 351 246 [53.5%] female) (Table 1). After propensity score matching, 58 629 hospitalization episodes were included for each group (Table 1). Prematched and postmatched characteristics are summarized between patients with and without ADRD (Table 1).

**Figure 1.**
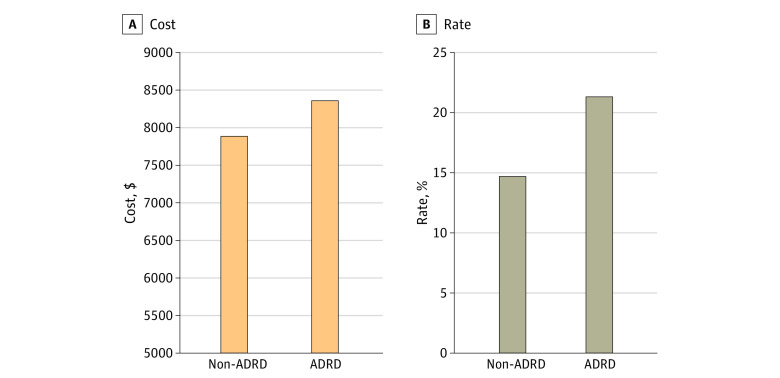
Readmission Rates and Costs Among Propensity-Matched Cohorts of Hospitalized Patients With and Without Alzheimer Disease and Related Dementias (ADRD), 2012-2017 Cost of readmission is based on direct winsorized standardized payments among patients with readmission. Data are from the Michigan Value Collaborative.

Figure 1 shows the mean risk-adjusted 30-day readmission rate and winsorized readmission-specific payment. The rates of readmission were 21.5% (95% CI, 21.2%-21.8%) for patients with ADRD and 14.7% (95% CI, 14.4%-15.0%) for patients without ADRD (difference, 6.75 percentage points; 95% CI, 6.31-7.19 percentage points). The cost of 30-day readmission was similarly $467 higher (95% CI for difference, $289-$645) among patients with ADRD ($8378; 95% CI, $8263-$8494) compared with those without ($7912; 95% CI, $7776-$8047).

**Figure 2.**
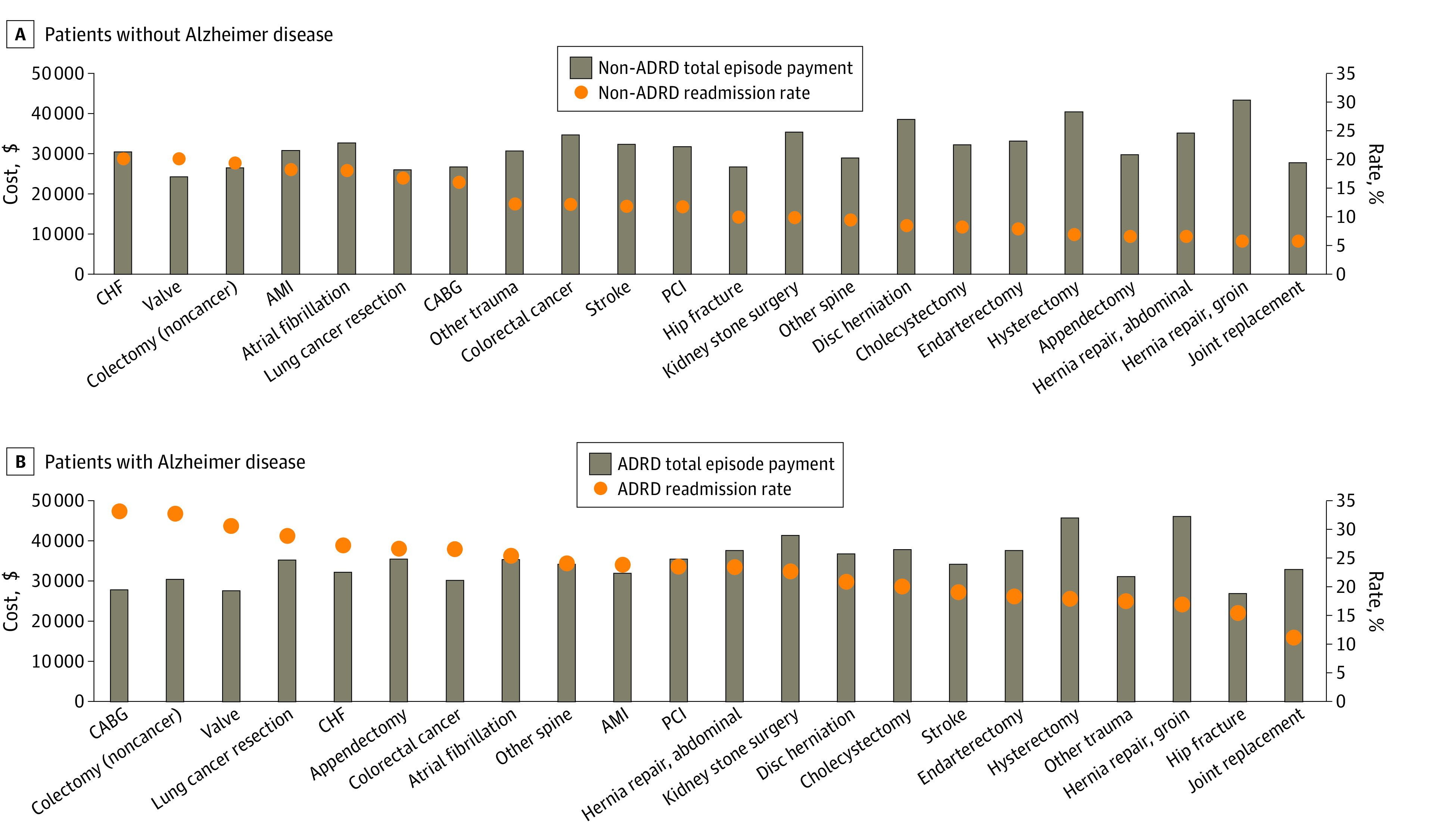
Readmission Rates and Total Episode Costs Among Propensity-Matched Cohorts of Hospitalized Patients With and Without Alzheimer Disease and Related Dementias (ADRD), Stratified by Service Line, 2012-2017 Cost of readmission is based on direct winsorized standardized payments among patients with readmission. Data are from the Michigan Value Collaborative, 2012-2017. AMI indicates acute myocardial infarction; CABG, coronary artery bypass graft; CHF, congestive heart failure; and PCI, percutaneous coronary intervention.

Figure 2 shows the mean, risk-adjusted, and winsorized 30-day total episode payments as well as the 30-day readmission rates across service lines. Readmission rates for patients with ADRD were ordered by descending rates across service lines. Across all 22 service lines, readmission rates were substantially higher among patients with ADRD (ranging from 11.2% for joint replacement to 33.2% for coronary artery bypass graft) compared with patients without (ranging from 5.9% for joint replacement to 20.3% for congestive heart failure). Across all service lines, the mean total 30-day episode cost of care was $2794 higher for patients with ADRD compared with those without ($22 371 vs $19 578; 95% CI of difference, $2668-$2919). Total 30-day episode payments include 30-day readmission and postdischarge payments (including home health, inpatient rehabilitation, and skilled nursing facility). Postdischarge payments were substantially higher for patients with ADRD than those without ADRD, driven by substantial differences in skilled nursing facility payments (eTable 2 and eFigure 2 in Supplement 1). In some service lines, such as hip and knee joint replacements, cholecystectomies, and colectomies with benign indications, the episode cost differences were substantially higher for patients with ADRD. The total episode costs for patients with and without ADRD and component payments summarized across all service lines are reported in eTable 2 in Supplement 1.

Table 2 gives the readmission costs across service lines. Except for acute myocardial infarction and hip fracture, no significant differences in readmission costs were found between patients with and without ADRD. Patients without ADRD had a $505 higher cost of readmission than patients with ADRD (95% CI for difference, $49-$961). On the other hand, readmission costs for hip fracture were higher by $766 for patients with ADRD than those without (95% CI for difference, $54-$1478).

**Table 2.** Total 30-Day Readmission Costs Across All Service Lines for Patients With and Without ADRD^a^

Service line	Cost, $ (95% CI)		
	With ADRD	Without ADRD	Difference
AMI	6735 (6446 to 7023)	7240 (6879 to 7601)	−505 (−49 to −961)
Appendectomy	10 069 (6810 to 13 327)	9666 (6887 to 12 445)	402 (7419 to −6615)
Atrial fibrillation	7043 (6732 to 7355)	6861 (6472 to 7251)	182 (676 to −312)
CABG	8248 (6825 to 9671)	8674 (6431 to 10 917)	−426 (2111 to −2964)
CHF	5997 (5835 to 6159)	6004 (5813 to 6195)	−7 (243 to −256)
Cholecystectomy	11 137 (9839 to 12 436)	11 350 (9302 to 13 398)	−213 (2253 to −2679)
Colectomy (noncancer)	6653 (5975 to 7331)	5980 (5118 to 6842)	673 (1773 to −427)
Colorectal cancer	8810 (7264 to 10 356)	11 522 (8843 to 14 201)	−2712 (142 to −5566)
Disc herniation	13 894 (7524 to 20 264)	15 769 (4130 to 27 408)	−1875 (9567 to −13316)
Endarterectomy	11 063 (9245 to 12 881)	10 094 (7718 to 12 471)	969 (4034 to −2097)
Hernia repair			
Abdominal	10 702 (8684 to 12 719)	9314 (5564 to 13 064)	1388 (5897 to −3121)
Groin	18 428 (16 362 to 20 494)	17 940 (14 021 to 21 859)	488 (4691 to −3716)
Hip fracture	10 844 (10 396 to 11 293)	10 079 (9531 to 10 626)	766 (1478 to 54)
Hysterectomy	20 119 (17 278 to 22 959)	18 101 (12 190 to 24 013)	2017 (7856 to −3822)
Joint replacement	18 368 (16 976 to 19 760)	15 974 (14 031 to 17 916)	2395 (4805 to −16)
Kidney stone surgery	11 928 (10 627 to 13 229)	10 075 (7949 to 12 201)	1853 (4338 to −632)
Lung cancer resection	10 789 (6735 to 14 844)	6771 (4255 to 9288)	4018 (9694 to −1658)
Other spine procedures	12 834 (11 579 to 14 089)	11 838 (9620 to 14 057)	996 (3437 to −1445)
Other trauma	9523 (9258 to 9788)	9221 (8898 to 9544)	302 (719 to −115)
PCI	9588 (8416 to 10 761)	8677 (7076 to 10 279)	912 (2938 to −1115)
Stroke	9721 (9398 to 10 044)	9619 (9210 to 10 028)	102 (621 to −416)
Valve	7484 (6461 to 8507)	6384 (5179 to 7589)	1100 (2677 to −477)

Abbreviations: ADRD, Alzheimer disease and related dementias; AMI, acute myocardial infarction; CABG, coronary artery bypass graft; CHF, congestive heart failure; PCI, percutaneous coronary intervention.

^a^ Data are from the 2012-2017 Michigan Value Collaborative matched cohorts of hospitalized patients with and without ADRD. Significance was assessed when 95% CIs of differences did not cross zero. All costs are price standardized and inflation adjusted using the Consumer Price Index to year 2017 dollars.

Table 3 gives the procedure-specific readmission costs, rates, and total facility episode payments (eg, only readmission and initial [index] hospitalization payments) with simple decomposition to reflect differences between patients with and without ADRD. In the propensity-matched cohort, when accounting for differences in readmission rates and costs between patients with and without ADRD, there was a range in total difference in total episode payments from $275 to $2333 across all procedure service lines considered.

**Table 3.** Decomposition of Costs Between Patients With and Without ADRD for Index Inpatient Hospitalization and Readmission Payments on the Propensity-Matched Cohort, Attributing Differences in Cost After Accounting for Differences in Procedure-Specific Readmission Rate, Total Episode Payments, and Cost of Readmissions^a^

Condition	Readmission						Index hospitalization payments			Decomposition			
	Rate, %			Cost, $									
	Absolute difference	Without ADRD	With ADRD	Absolute difference	Without ADRD	With ADRD	Without ADRD	With ADRD	Absolute difference	Total cost, $		Difference accounting for readmission rate difference revised^d^	Difference attributable to difference in readmission rate and cost^e^
										With ADRD^b^	Without ADRD^c^		
AMI	−5	18.4	23.9	505	7240	6735	12 879	12 667	−213	14 275	14 213	62	275
Appendectomy	−20	6.7	26.7	−402	9666	10 069	12 065	14 792	2727	17 477	12 715	4762	2036
Atrial fibrillation	−7	18.3	25.5	−182	6861	7043	12 705	12 687	−18	14 483	13 959	524	542
CABG	−17	16.2	33.2	426	8674	8248	14 226	13 469	−756	16 208	15 632	576	1332
CHF	−7	20.3	27.3	7	6004	5997	12 548	12 563	15	14 197	13 766	431	416
Cholecystectomy	−12	8.4	20.1	213	11 350	11 137	13 048	15 235	2187	17 470	14 000	3470	1283
Colectomy (noncancer)	−13	19.6	32.8	−673	5980	6653	13 004	14 897	1893	17 078	14 175	2903	1010
Colorectal cancer	−14	12.3	26.6	2712	11 522	8810	15 497	13 554	−1943	15 900	16 918	−1018	925
Disc herniation	−12	8.6	20.9	1875	15 769	13 894	15 643	16 896	1253	19 804	16 994	2810	1557
Endarterectomy	−10	8.1	18.4	−969	10 094	11 063	11 874	14 884	3010	16 917	12 688	4229	1219
Hernia repair													
Abdominal	−17	6.7	23.5	−1388	9314	10 702	15 171	14 979	−192	17 491	15 794	1696	1889
Groin	−11	5.9	16.9	−488	17 940	18 428	13 365	13 711	346	16 832	14 423	2409	2063
Hip fracture	−5	10.1	15.5	−766	10 079	10 844	12 259	12 851	592	14 529	13 275	1254	662
Hysterectomy	−11	7.1	18.0	−2017	18 101	20 119	16 328	14 382	−1946	17 993	17 606	387	2333
Joint replacement (knee and hip)	−5	5.9	11.2	−2395	15 974	18 368	12 544	13 539	995	15 596	13 485	2111	1116
Kidney stone surgery	−13	10.0	22.8	−1853	10 075	11 928	13 412	15 766	2354	18 479	14 417	4062	1708
Lung cancer resection	−12	16.9	29.0	−4018	6771	10 789	13 307	17 418	4111	20 541	14 451	6090	1979
Other spine	−15	9.6	24.1	−996	11 838	12 834	12 577	15 556	2979	18 651	13 709	4942	1963
Other trauma	−5	12.4	17.5	−302	9221	9523	13 639	13 594	−45	15 261	14 781	479	524
PCI	−12	11.9	23.6	−912	8677	9588	13 177	14 103	927	16 365	14 208	2158	1231
Stroke	−7	12.0	19.1	−102	9619	9721	13 917	14 431	513	16 291	15 070	1222	708
Valve	−10	20.3	30.6	−1100	6384	7484	13 228	14 224	996	16 516	14 523	1992	996

Abbreviations: ADRD, Alzheimer disease and related dementias; AMI, acute myocardial infarction; CABG, coronary artery bypass graft; CHF, congestive heart failure; PCI, percutaneous coronary intervention.

^a^ All costs are price standardized and inflation adjusted using the Consumer Price Index to year 2017 dollars. This decomposition includes the facility index payments and the facility readmission payments.

^b^ Total index hospitalization costs for patients with ADRD plus readmission cost for patients with ADRD.

^c^ Total index hospitalization costs for patients without ADRD plus readmission cost for patients without ADRD.

^d^ Difference accounting for readmission rate difference revised is calculated as index hospitalization costs for patients with ADRD minus index hospitalization costs for patients without ADRD plus readmission costs for patients with and without ADRD.

^e^ Difference attributable to differences in readmission rate and cost.

## Discussion

This cohort study underscores 3 key findings pertaining to 30-day readmission rates and episode costs of care comparing patients with and without ADRD. First, large variation exists in readmission rate between and within patients with and without ADRD across various service lines (ie, medical and surgical procedures). Second, across all service lines, the mean rate of 30-day readmission was 6.8 percentage points higher for patients with ADRD than those without; however, the mean cost of readmission was comparable between the 2 groups. Third, the mean total 30-day episode cost of hospitalization is higher among patients with ADRD compared with patients without, driven by a large proportion in postdischarge costs, differences in readmission rates, and cost of readmission for each procedure.

Considerable county-level variation in readmission might be the manifestation of existing social and environmental disparities. County-level socioeconomic characteristics and the number of primary care and general internal physicians vs the number of hospitals are strong factors associated with readmission and potentially preventable hospitalizations.^20^ People in poorer communities have comparatively lower access to primary care physicians for required follow-up appointments after discharge. In addition, they have challenges with access to transportation for food and medicine they need for daily life.^21^ In Michigan, for example, Wayne, Saginaw, and Genesee counties are among those with the highest readmission rates for patients with (20%-27%) and without ADRD (9%-17%). According to the 2019 County Health Rankings State Report for Michigan,^22^ counties with the highest rate of readmission are also among the most deprived counties in measures of access to care, quality of care, social and economic factors (including educational level, employment, income, social and family support, and community safety), and physical environment (including housing and transition).^23^ Although we could not formally test this possibility using formal measures of social determinants of health, our findings support prior research^20^ on strong association between social factors at a community level and readmission rates.

Since 2012, hospitals have been a primary target to reduce readmission. Thus, according to the Hospital Readmissions Reduction Program (HRRP),^24^ the CMS penalizes hospitals with excessive readmission rates for certain conditions. For patients with ADRD, the implementation of HRRP does not account for the highly heterogeneous patient population, including disparate outcomes (ie, delirium, cognitive decline, institutionalization, and premature death).

Our findings indicate a substantially higher risk of readmission among patients with ADRD compared with the matched cohort of patients without ADRD. Postdischarge adverse outcomes, such as delirium, frailty, institutionalization, and premature death, are substantially worse for patients with ADRD than for the general geriatric population.^25^ Number of hospitalizations, 30-day readmission, and cost of postacute care for patients with ADRD are higher than in the general geriatric population.^12,26^ Although approximately 40% of older hospitalized patients are cognitively impaired, most hospitals are ill-equipped to minimize the risk of readmission and non–value-added health care spending for this increasing population. Hospital discharges often come without adequate preparation for complex case management needs of patients with ADRD and their caregivers. It follows that people with ADRD are highly vulnerable and very resource intensive, with substantially higher readmission risk and elevated rates of other adverse health events. Depending on severity of ADRD, physicians might forgo inpatient procedures, unless they add value to a patient’s quality of life. Hospitalization may expedite dementia and cognitive decline and elevate the risks of long-term institutionalization and premature death among patients with ADRD.^27^ Furthermore, preoperative assessment should be considered for advanced cases of ADRD to judiciously assess whether hospitalization would further exacerbate downstream health conditions for the patient.^28^

Finally, our findings indicate that 30-day total episode payments were substantially higher for patients with ADRD than patients without, associated mainly with substantial differences in skilled nursing facility payments. Approximately 34% of hospitalized patients with ADRD are discharged to skilled nursing facilities, where 59% of them are readmitted to a hospital within a short time frame.^29^ Just as the CMS implemented strategies to reduce readmissions for hospitals with HRRP, they similarly initiated a program that aimed to reduce excess transfer rates to hospitals for skilled nursing facilities.^30,31^ Perhaps these strategies would be more successful if they accounted for patient case mix or specific resource-intensive patient populations.

### Strengths and Limitations

This study has several noteworthy strengths. We examined a large sample of patient episodes across 77 hospitals in Michigan. To our knowledge, this is the first study to compare patients with and without ADRD that analyzes readmission rate, costs, and total 30-day episode costs of care across service lines. Because we covered almost all hospitals in Michigan, our findings are generalizable and in agreement with prior research.^32^ Another strength of this study is the specificity of covariates used for propensity matching patients with and without ADRD. For example, instead of using composite measures of patient acuity, such as the Charlson and Elixhauser comorbidity indexes, we matched on all 79 Hierarchical Condition Categories to account for each clinical condition the patients present with at the time of the index hospitalization. This study helps illuminate the extent to which differences in rates and cost of readmission across procedure groups drive differences in total episode payments for patients with ADRD compared with their matched controls. Finally, on the basis of the strength of our unique data set, our analytical approach addressed price standardization and risk adjustment to ensure appropriate cost comparisons across the state and patient settings.

Our study also has several limitations. First, due to well-documented difficulties in the clinical diagnosis of ADRD, both clinical and claims data are limited in correctly identifying all patients with ADRD.^33^ We used *ICD-9-CM* and *ICD-10-CM* codes to identify patients with ADRD. To address this inherent limitation of the data, we propensity matched our cases and controls based on their demographic and clinical characteristics to reduce potential selection bias.^34,35^ Second, more detailed sociodemographic information about our patients, such as race, ethnicity, and income, were not available to fully adjust and account for in our analysis. If the data set included direct measures of key social determinants of health, we could have more carefully accounted for this key factor. In general, certain comorbidities for adjustment might be undercoded in claims data. At the time of this study, county-level or other geographic identifiers that would enable additional attributes were not available for matching and could represent unobserved confounding that was not included in the matching of patients with and without ADRD.

## Conclusions

This cohort study found that patients with ADRD had higher readmission rates and overall episode costs than patients without ADRD. Avoidable hospitalization undermines the quality of life and longevity, possibly increasing the risk for adverse events for patients with ADRD.^25^ Although more research is required to understand the variance across service lines in readmission risk between patients with and without ADRD, measures should be taken to ensure patient fitness for inpatient care.

## Notes

Supplement 1. **eAppendix 1.** Michigan Value Collaborative (MVC) Price Standardization Methodology **eAppendix 2.** Risk Adjustment Methodology **eAppendix 3.** Decomposition Framework **eFigure 1.** Schematic Flow Diagram of Patient Sample Size **eFigure 2.** Risk Adjusted, Winsorized Payments for Propensity Matched Cohort of Patients With and Without ADRD (2012-2017) in the Michigan Value Collaborative, Stratified by Service Line and Inflation-Adjusted to 2017 Dollars **eTable 1.** ICD-9 and ICD-10 Codes for ADRD **eTable 2.** Risk Adjusted/Winsorized/Standardized and Inflation Adjusted Total Episode Payment Categories Between ADRD and Non-ADRD Patients

Supplement 2.
Data Sharing Statement
