# Supplementary material for: Readmission Rates and Episode Costs for Alzheimer Disease and Related Dementias Across Hospitals in a Statewide Collaborative
Source: JAMA Netw Open. 2023 Mar 16;6(3):e232109. doi: 10.1001/jamanetworkopen.2023.2109 (PMC10020873; doi:10.1001/jamanetworkopen.2023.2109)
Supplement: Supplement 1. — eAppendix 1. Michigan Value Collaborative (MVC) Price Standardization Methodology eAppendix 2. Risk Adjustment Methodology eAppendix 3. Decomposition Framework eFigure 1. Schematic Flow Diagram of Patient Sample Size eFigure 2. Risk Adjusted, Winsorized Payments for Propensity Matched Cohort of Patients With and Without ADRD (2012-2017) in the Michigan Value Collaborative, Stratified by Service Line and Inflation-Adjusted to 2017 Dollars eTable 1. ICD-9 and ICD-10 Codes for ADRD eTable 2. Risk Adjusted/Winsorized/Standardized and Inflation Adjusted Total Episode Payment Categories Between ADRD and Non-ADRD Patients [file jamanetwopen-e232109-s001.pdf]

Supplementary Online Content

Kamdar N, Syrjamaki J, Aikens JE, Mahmoudi E. Readmission rates and episode costs for Alzheimer disease and related dementias across hospitals in a statewide collaborative. *JAMA Netw Open*. 2023;6(3):e232109. doi:10.1001/jamanetworkopen.2023.2109

eAppendix 1. Michigan Value Collaborative (MVC) Price Standardization Methodology

eAppendix 2. Risk Adjustment Methodology

eAppendix 3. Decomposition Framework

eFigure 1. Schematic Flow Diagram of Patient Sample Size

eFigure 2. Risk Adjusted, Winsorized Payments for Propensity Matched Cohort of Patients With and Without ADRD (2012-2017) in the Michigan Value Collaborative, Stratified by Service Line and Inflation-Adjusted to 2017 Dollars

eTable 1. ICD-9 and ICD-10 Codes for ADRD

eTable 2. Risk Adjusted/Winsorized/Standardized and Inflation Adjusted Total Episode Payment Categories Between ADRD and Non-ADRD Patients

This supplementary material has been provided by the authors to give readers additional information about their work.

© 2023 Kamdar N et al. *JAMA Network Open.*

eAppendix 1. Michigan Value Collaborative (MVC) Price Standardization Methodology

The MVC claims data and analytic products leverage a process that price standardizes and risk adjusts the episode payments. This process is meant to account for price variations attributable to negotiated contracts, inflation, wage index, geographic region, payer, and/or hospital characteristics. These prices are assigned based on the Medicare fee-for-service data per their schedule. The method of standardizing payments are a measure of utilization instead of actual cost.

Price standardization is performed in a manner that divides data into three parts:

1.) Inpatient facility claims

2.) Other facility claims

3.) Professional claims as a single group

Inpatient Facility Claims

There are three inpatient facility claim payment amounts that are estimated. First, there is use of the Diagnosis Related Group (DRG) base payment, which assigns an average price based on Medicare data using the most recent relative weight (DRG weight) over time. The five data elements include patient sex, age, patient discharge disposition, ICD9/10 diagnosis codes, and ICD9/10 procedure codes).

Second, outlier payments are assigned separately from the DRG base payment to providers to compensate for patients that are particularly complicated (and ultimately very high cost patients). This occurs when the level of treatment greatly exceeds the expected average payment). These

© 2023 Kamdar N et al. *JAMA Network Open.*

are usually identified when the hospitalization length of stay for that specific DRG is substantially larger than the average. For the current price standardization approach, an outlier payment is calculated as $2500/day x each day over the length-of-stay threshold as defined by the DRG classification for that group.

Finally, transfer payments are estimated when a patient is transferred from one hospital to another, where both the initial hospital as well as the transfer hospital bill for an inpatient admission are billed separately. Transfer payments are assigned for the inpatient stay occurring at the destination hospital (e.g. at the end of the transfer, destination hospital). If no transfers had occurred, these were assigned a 0.

Post-Acute Care Claims

Inpatient Rehabilitation Claims

All inpatient rehabilitation claims are priced based on DRG. Inpatient rehabilitation claims are pro-rated in the episode period; therefore, only the fraction of days during the episode period (e.g. 30 or 90-day episodes) will be included.

Skilled Nursing Facility (SNF) Claims

Medicare uses a Patient Driven Payment Model (PDPM)[1], and MVC implements this per diem payment across conditions, payers, and years. SNF payment variation will be fully due to utilization and length of stay. SNF stays that extend past the episode length (e.g. 30 and 90-day) will be pro-rated.

Outpatient Rehabilitation Claims

All outpatient rehabilitation claims are priced based on CPT codes.

© 2023 Kamdar N et al. *JAMA Network Open.*

Home Health (HH) Claims

All home health claims are standardized using predetermined base payments under the Prospective Payment System (PPS)[2] from Medicare. Base payments are adjusted according to characteristics in the Home Health Resource Groups (HHRG), including different patient health conditions and patient care needs. HH payments are based on the code rates and length of service. Medicare payment policies dictate that if the patient receives four or fewer visits during a 60-day episode, services are paid using a standardized per visit payment using the Healthcare Common Procedure Coding System (HCPCS) code. Medicare refers to this payment adjustment as a Low Utilization Payment Adjustment (LUPA). For HH claims that have more than four visits during the 60-day episode, the payments are calculated based on the HHRG code per the Non-LUPA payment schedule; therefore, different payment schedule than the low utilization payments. All claims in MVC are standardized according to Medicare's rules.

Emergency Department (ED) Claims

ED claims are priced based on the CPT codes. MVC standardizes the method for identifying the ED visits to account for hospital differences. Typically, ED visits that directly precede hospital admissions are billed as a claim line within that hospital admissions. Therefore, there would be no separate facility payment with that ED visit since the hospital stay is paid based on DRG. Critical Access Hospitals (CAHs) bill separately for ED visits and that ultimately results in payment and rate differences. MVC does not price ED claims at CAHs that occur on the same date as an index admission.

Professional Claims

Each professional claim has an associated CPT code, quantity, and unit that are used to calculate the total payment. CPT modifiers are used to increase or decrease the standard payment for that claim.

© 2023 Kamdar N et al. *JAMA Network Open.*

eAppendix 2. Risk Adjustment Methodology

MVC performs risk adjustment using observed (O)-to-expected (E) ratios (O/E). The numerator of the ratio represents the aggregate of all observed payments for a particular hospital. The denominator represents the aggregate of all the expected payments. The ratio of these two quantities is multiplied by the overall state-wide expected mean payment to estimate the risk adjusted payment for that hospital. Expected payments are estimated for each clinical condition or service line (e.g. Acute Myocardial Infarction, etc.), and payment component (e.g. total episode payments, etc.). Model estimation is used, with required variables including age, gender, insurance type, high prior six-month payments, and end-stage renal disease. Non-required variables leverage the 79 comorbidities based on the Hierarchical Condition Categories (HCCs) and condition-specific risk adjusters. Model specification requires that all candidate variables are individually tested using univariate regression models to examine if they predict payment. Those variables whose p-value in univariate analysis of <0.10 are retained for future model specifications. All retained variables are then included in a multivariable regression model with a p-value cutoff of <0.05.

© 2023 Kamdar N et al. *JAMA Network Open.*

eAppendix 3. Decomposition Framework

To estimate the difference in costs after propensity score matching on observable characteristics, we considered a simple decomposition framework to further tease out differences attributable to readmission costs and rates between ADRD and non-ADRD patients. Therefore, the following approach was taken to estimate these differences.

We let D be the 30-day total episode payment difference between ADRD and non-ADRD patients, and TCA and TCN be the total 30-day episode payments between ADRD (A) and non-ADRD (N), respectively.

D = TCA – TCN (Eq. 1)

For each of ADRD and non-ADRD, we can split the total 30-day payments into two components:

TCA = HA + fRA (Eq. 2)

TCN = HN + gRN (Eq. 3)

Where HA and HN are the initial hospitalization payments in the episode for each of ADRD and non-ADRD patients, respectively. We let RA and RN and f and g represent the average cost of readmissions in ADRD (RA) and non-ADRD (RN) and the observed rates (f) and (g) for ADRD and non-ADRD, respectively.

Therefore, by substitution:

D = TCA – TCN = HA + fRA – (HN + gRN) = HA – HN + fRA – gRN. (Eq. 4)

© 2023 Kamdar N et al. *JAMA Network Open.*

This enables estimation of the components of cost attributable to the readmission cost and rate difference between ADRD and non-ADRD patients. Furthermore, expansion of Eq. 4 yields:

$$D = HA - HN + \left(\frac{f+g}{2}\right)(RA - RN) + \left(\frac{RA+RN}{2}\right)(f - g)$$

This allows fRA – gRN to be composed into terms representing $\left(\frac{f+g}{2}\right)(RA - RN)$ which is the product of the procedure-specific average rate of readmission multiplied by the average difference in readmission cost, and $\left(\frac{RA+RN}{2}\right)(f - g)$ represents the procedure-specific average readmission cost multiplied by the difference in readmission rates between both groups. All costs compared were price standardized, inflation-adjusted, and risk-adjusted prior to performing these comparisons.

References:

1. Patient Driven Payment Model - CMS, https://www.cms.gov/Medicare/Medicare-Fee-for-Service-Payment/SNFPPS/PDPM
2. Home Health PPS | CMS, https://www.cms.gov/Medicare/Medicare-Fee-for-Service-Payment/HomeHealthPPS

© 2023 Kamdar N et al. *JAMA Network Open.*

eFigure 1. Schematic Flow of Cohort Selection

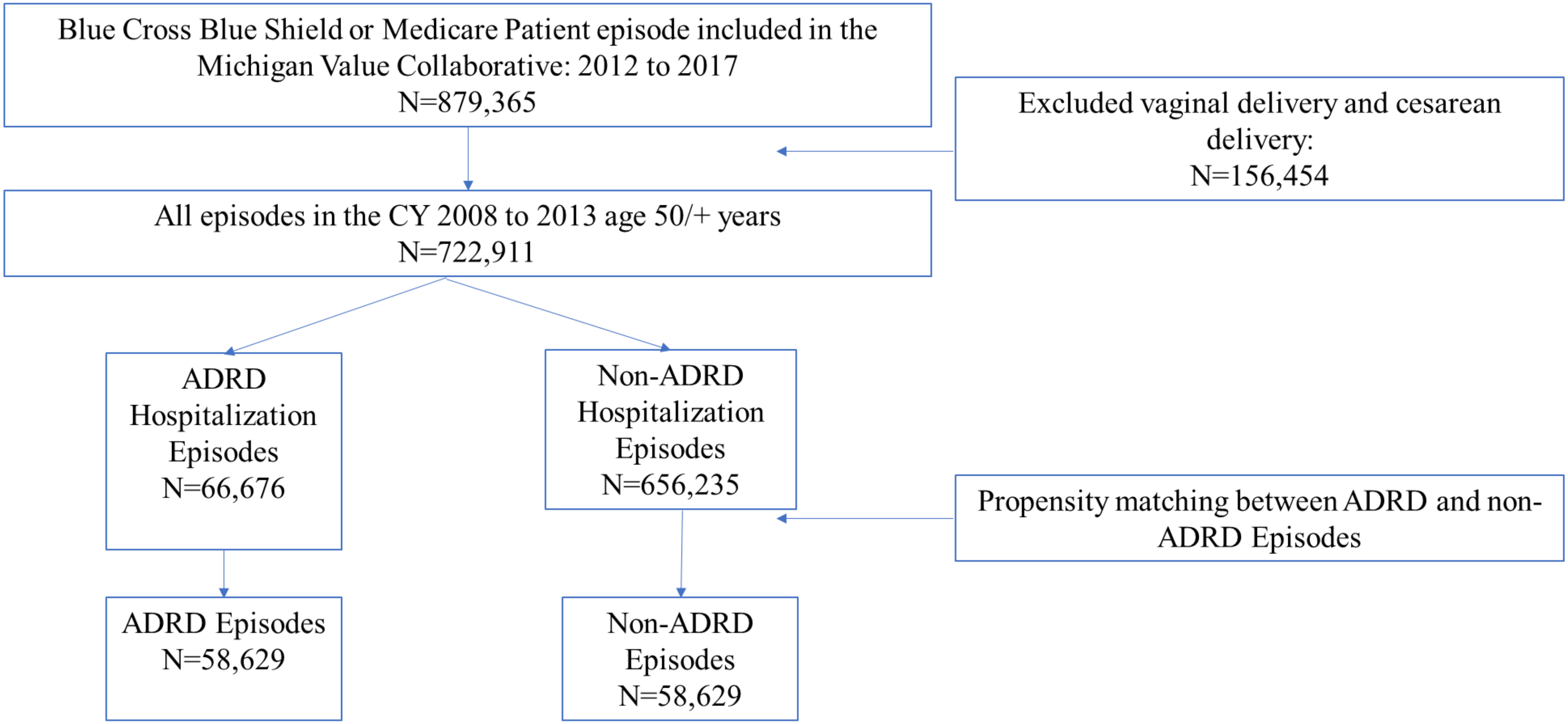

© 2023 Kamdar N et al. *JAMA Network Open.*

eFigure 2. Risk Adjusted, Winsorized Hospital Episode Payments for Propensity Matched Cohort of Non-ADRD and ADRD Patients, stratified by service line and inflation-adjusted to 2017 dollars

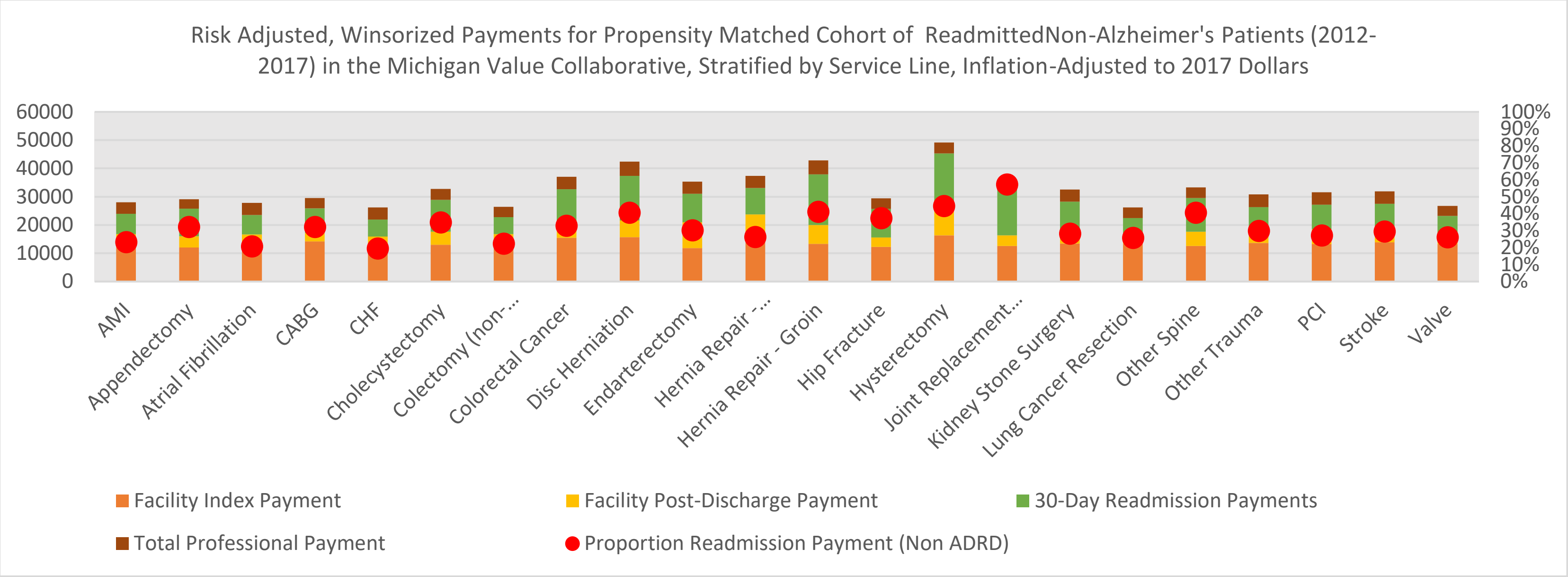

© 2023 Kamdar N et al. *JAMA Network Open.*

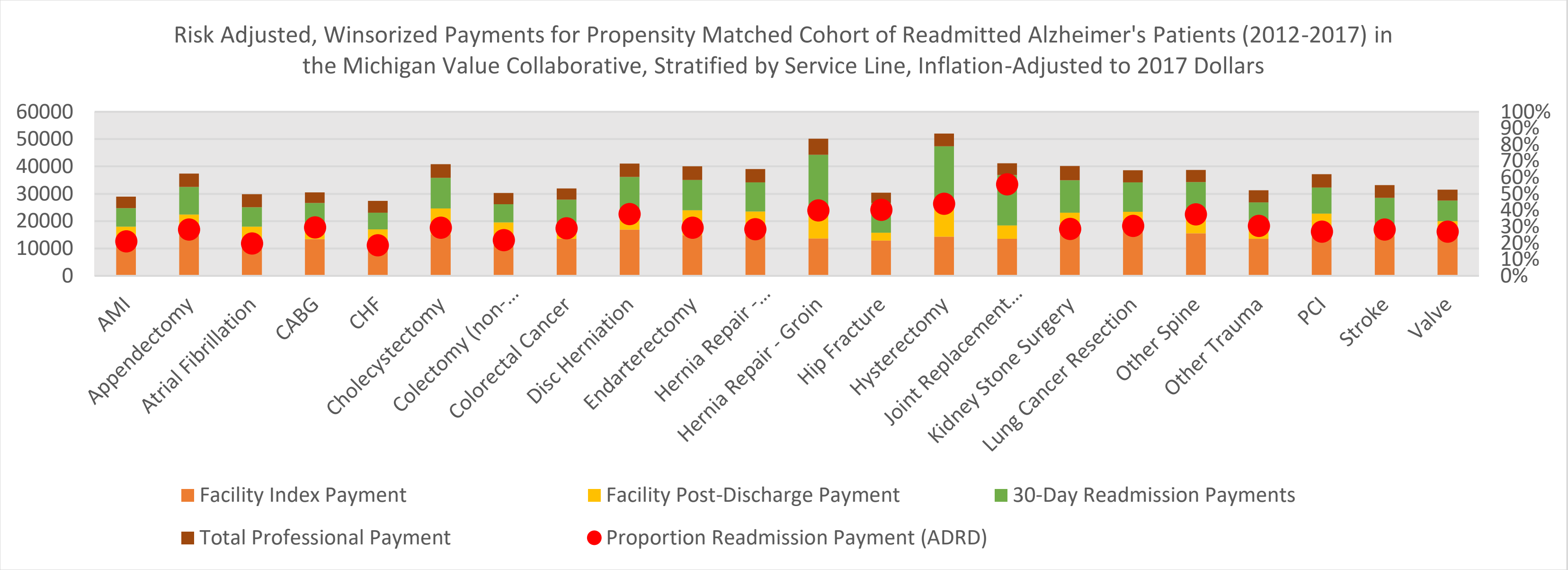

Note: The facility index payment, total professional payment, facility post-discharge payment, and 30-day readmission payment reflect majority of the broad component payments that comprise the 30-day total episode payments. Total 30-day episode payments will be larger than the per-procedure totals indicated here. Figure 2 reflects the total 30-day episode payment including these four payment components as well as all other payments for each procedure.

© 2023 Kamdar N et al. *JAMA Network Open.*

eTable 1. ICD-9 and ICD-10 Diagnosis codes for Alzheimer's Disease and Related Dementia

Diagnosis code	*Description*	*ICD Version*
290	DEMENTIAS	9
2900	SENILE DEMENTIA, UNCOMPLICATED	9
29010	PRESENILE DEMENTIA, UNCOMPLICATED	9
29011	PRESENILE DEMENTIA WITH DELIRIUM	9
29012	PRESENILE DEMENTIA W/DLUSIONL FTUR	9
29013	PRESENILE DEMENTIA W/DPRSV FEATURES	9
29020	SENILE DEMENTIA W/DLUSIONL FEATURES	9
29021	SENILE DEMENTIA W/DPRSV FEATURES	9
2903	SENILE DEMENTIA WITH DELIRIUM	9
2904	VASCULAR DEMENTIA	9
29040	VASCULAR DEMENTIA UNCOMPLICATED	9
29041	VASCULAR DEMENTIA WITH DELIRIUM	9
29042	VASCULAR DEMENTIA WITH DELUSIONS	9
29043	VASCULAR DEMENTIA W/DEPRESSED MOOD	9
29282	DRUG-INDUCED PERSISTING DEMENTIA	9
29410	DEMENTIA CCE W/O BEHAV DISTURB	9
29411	DEMENTIA CCE W/BEHAV DISTURBANCES	9
29420	DEMENTIA UNS W/O BEHAV DISTURBANCE	9
29421	DEMENTIA UNS W/BEHAVORL DISTURBANCE	9
3310	ALZHEIMERS DISEASE	9
F01	VASCULAR DEMENTIA	10
F0150	VASC DEMENTIA W/OUT BEHAVIORAL DIST	10
F0151	VASC DEMENTIA WITH BEHAVIORAL DIST	10
F02	DEMENTIA OTH DISEAS CLASS ELSW	10
F0280	DEMENTIA OTH DISEAS W/O BHVRL DIST	10
F0281	DEMENTIA OTH DISEASE W/BEHAVRL DIST	10
F03	UNSPECIFIED DEMENTIA	10
F0390	UNS DEMENT W/O BEHAVIORAL DIST	10
F0391	UNS DEMENT W/BEHAVIORAL DISTURBANCE	10
G300	ALZHEIMERS DISEASE WITH EARLY ONSET	10
G301	ALZHEIMERS DISEASE WITH LATE ONSET	10
G309	ALZHEIMERS DISEASE UNSPECIFIED	10

© 2023 Kamdar N et al. *JAMA Network Open.*

eTable 2. Risk Adjusted/Winsorized/Standardized and Inflation-Adjusted total Episode Payment Categories Between ADRD and Non-ADRD Patients

	Post-Matched Cohort		
**Risk Adjusted/Winsorized/Standardized Payment Category, Mean (95% CI)**	**Non-Alzheimer's/Dementia (N=58,629)**	**Alzheimer's/Dementia (N=58,629)**	**Absolute Difference (95% CI)**
Total 30-day Episode Payment	19578 (19493, 19662)	22371 (22278, 22464)	2794 (2668, 2919)
Total Professional Payment	2598 (2585, 2610)	2879 (2866, 2893)	282 (263, 300)
Total Facility Index Base Payment	12178 (12138, 12218)	12560 (12518, 12601)	382 (324, 440)
Total Facility Index Payment	12417 (12372, 12461)	12807 (12761, 12854)	391 (326, 455)
Total Facility Post-Discharge Payment	3278 (3245, 3311)	4554 (4512, 4596)	1276 (1223,1330)
Total 30-day Readmission Payments	1166 (1135, 1196)	1800 (1763, 1837)	634 (586, 682)
Total Outpatient Home Health Payments	398 (392, 405)	378 (371, 385)	21 (11, 30)
Total Facility Inpatient Rehabilitation Payments	378 (366, 390)	345 (333, 357)	33(15, 50)
Total Skilled Nursing Facility Payments	1564 (1539, 1588)	2725 (2690, 2760)	1161 (1118, 1204)

© 2023 Kamdar N et al. *JAMA Network Open.*
