# Supplementary material for: Readmission Rates and Episode Costs for Alzheimer Disease and Related Dementias Across Hospitals in a Statewide Collaborative
Source: JAMA Netw Open. 2023 Mar 16;6(3):e232109. doi: 10.1001/jamanetworkopen.2023.2109 (PMC10020873; doi:10.1001/jamanetworkopen.2023.2109)
Supplement: Supplement 2. — Data Sharing Statement [file jamanetwopen-e232109-s002.pdf]

Data Sharing Statement

Kamdar. Readmission Rates and Episode Costs for Alzheimer Disease and Related Dementias Across Hospitals in a Statewide Collaborative. *JAMA Netw Open*. Published March 16, 2023. doi:10.1001/jamanetworkopen.2023.2109

Data
Data available: No

Additional Information
Explanation for why data not available: Per our agreement with Blue Cross Blue Shield through the Michigan Value Collaborative, we are unable to make available the administrative claims and episode files used for the derivation of the analysis. We can, however, make available the code/algorithms or other supporting documentation.
